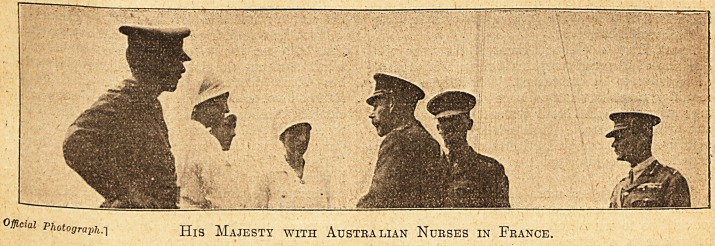# Matrons in Council: Sparks from a Nurse's Anvil

**Published:** 1917-08-04

**Authors:** 


### THE MATRONS' AND SISTERS' DEPARTMENT.

*Official Photograph.*] His Majesty with Australian Nurses in France.

### MATRONS IN COUNCIL.

### SPARKS FROM A NURSE'S ANVIL.

**[We have received the following article from a trained nurse of high character and many years experience. One of the great matrons of Great Britain shares our view that what the nurse says is quite in order and is just what many matrons are aiming at. We are encouraged therefore to hope that its publication may produce a full discussion on the points raised and prove topical and helpful.—Ed. "The Hospital."]**

In these days of reconstruction, when all the world—*i.e.* all the really thinking world—is silently asking themselves these two questions, "As an individual, how do I stand from the point of view of my standard?"; "As a member of my trade or profession, what is there in it which requires attention from the point of view of improvement?" the time is ripe for matrons to pass themselves in review. Theirs is a post of almost exceptional opportunities—as indeed is the position of every educational head—the world looks to them to send out the best quality procurable with which to carry on, to uphold traditions old and new, and create yet others. Florence Nightingale's idea was that nursing should be in the hands of educated women, but either the true meaning of education has been misunderstood, or else there has been a considerable divergence from her ideal.

Making all due allowance for diversity of ideas on the subject, it may be conceded that the whole point of education is to develop *quality*—there is the Quality of Ideal and the Quality of Performance. An Ideal without Performance is like a Brain without a Body; Performance without an Ideal is like a Body without a Brain; the one is unpractical, the other coarse and low. So what we look to the matrons to do is to seek to combine in their educational methods these two things.

There has been a tendency of late years to push to the front the competitive examination method, the award of marks, etc.; these are all very well in their way, naturally something must be defined as a guarantee to the outer world that a certain apprenticeship has been served, but it is by no means the whole; there are such varieties of individual standards of performance, such great differences in brain development, that an examination standard cannot but be very limited and partial in its guarantee. We want more. We want a guarantee of an ever-broadening Ideal, with an increasing perception of equal values in varieties of Performance; altogether something more elastic. In the new scheme of the formation of a Royal educational establishment, why should not some new and better standard be adopted, embodying all there is of good in past methods, and adding thereto whatever practical methods seem useful according to the needs which have arisen out of present life-conditions, so to bring us more into line with the Dominions and America? These exceedingly practical and go-ahead people are not remaining content with their original conceptions, but continue to search for further improvements—our schools must do the same.

If this scheme is to be as far-reaching as possible, to be *really* Imperial in its ultimate decisions, why should not the proposed Council of the new College include, *e.g.*, the Matron-in-Chief of the Army, and one or more of her staff? Her experience, and that of her associates, would be of great benefit to the profession as a whole; on the other hand, many unnecessary conventions in the Army would tend to disappear. All over the world, in every possible sort of way, the lesson of co-operation, pooling of resources, and common assistance for the common benefit, are being learned, either willingly or under compulsion. It is greatly to be deplored that even now, after the close alliance of the past three years, during which Army and civilian nurses have worked together for a common purpose, sharing common difficulties and experiences, there still exists this feeling of separateness. Amongst soldiers this feeling has died out almost entirely, but it still lives very strongly in the nursing world. If this could be eradicated, if the heads of the Navy and Army nursing staff could be permanently associated with the educational body, a great step towards unity of ideal would be gained.

Apart from education, there is the very vital question of financial reorganisation in every branch of nursing to be considered. This is a matter requiring much tact and patience and very fair dealing; matrons should bestir themselves energetically and without delay. It is advisable that a consultative board, combining heads of every shade of experience, should organise to consider these questions.

We would urge a more democratic attitude on the part of matrons towards the rank and file; we would like to insist that being a matron does not mean one is a better nurse or more experienced. On the contrary, it very frequently happens that the nursing experience of matrons is shorter and less varied than that of the constant worker; the difference between them resolves itself really into a question of temperament. Some are better fitted to go down into the labour and stress of fighting, to do the work itself; others feel themselves more suited in the less exposed official region. It does not matter; both sides of life must be carried out, only let those fulfilling the official capacity remember that both are really level in value; all alike are fellow-students working at their little piece; each has some particular contribution available for common use and benefit. Wisdom is not the exclusive possession of officials; it is a universal gift, revealing itself in manifold ways. Their part is indicated in the name assumed by them—"Matron," from the same root as "Mater," Mother. It is to them the workers should look for comfort and sympathy what time they fall out for rest between labours; it is they who should stand between the nurse and the outside need, to see that work does not develop into drudgery which is stale and unprofitable alike to the worker and the work; it is they who should be alert to protect the workers' interests from both a health and a financial point of view.

Every woman to her part: to All equal Honour.

## Figures and Tables

**Figure f1:**